# Breast cancer in a teenage girl with BRCA mutation: A case report from a low middle-income country

**DOI:** 10.1016/j.ijscr.2022.107513

**Published:** 2022-08-13

**Authors:** Lubna M. Vohra, Danish Ali, Syeda Amrah Hashmi, Meher Angez

**Affiliations:** a Department of Surgery, The Aga Khan University, P.O. Box 3500, Stadium Road, Karachi 74800, Pakistan; b Dean's Clinical Research Fellow, The Aga Khan University, P.O. Box 3500, Stadium Road, Karachi 74800, Pakistan; c The Aga Khan University, P.O. Box 3500, Stadium Road, Karachi 74800, Pakistan

**Keywords:** BC, Breast cancer, HBOC, Hereditary breast and ovarian cancer, LMIC, Low middle-income country, BRCA, Breast cancer gene, NCCN, National Comprehensive Cancer Network, BRBC, BRCA-positive Breast Cancer, CT, Computed tomography, US, Ultrasound, SLN, Sentinel lymph node, BAEF, Balochistan Awami Endowment Fund, GPs, General practitioners, RRSO, Risk-reducing salpingoopherectomy, Breast cancer, Case report, BRCA mutation, Pediatric breast cancer, LMIC, Low middle-income country

## Abstract

**Introduction:**

A staggering majority of pediatric breast masses are benign (mostly fibroadenoma) and so a biopsy is not readily performed as it can potentially lead to a future breast disfigurement. However, this should not be standard practice as this can lead to a delayed diagnosis, and hence, the treatment of pediatric breast cancer (BC); this was also seen in our patient's scenario.

**Case history:**

Here, we report the case of the youngest known breast cancer patient in Pakistan, a 15-year-old girl. The right-sided breast lump which was diagnosed clinically as a fibroadenoma later turned out to be stage IIb pT3N0M0 metaplastic breast carcinoma with BRCA1 positivity and mutations in SMARCA4. Being young and unmarried, the patient and her family decided to opt for breast-conserving surgery with high-risk surveillance for breast and ovaries.

**Discussion:**

We believe that prophylactic surgeries can be delayed with strict surveillance and thorough counseling. As pediatric BC is linked to a less favorable prognosis, every young patient diagnosed with breast cancer and their family should undergo genetic testing. BC management should be handled by specialists in the field and doctors should be trained for initial diagnostics and timely referral of patients.

**Conclusion:**

It is important to improve our understanding of genetic predisposition and testing in lower-middle-income countries. Considering the changing global trends, we suggest that the utilization of genetic services is direly needed to improve preventative care for at-risk individuals with breast and other cancers.

## Highlights

• The case of the youngest known breast cancer patient in Pakistan.
• Genetic counseling should be readily offered in low middle-income countries.
• Triple assessment is a mandatory tool to diagnose a breast lump in any age group.
• Up-to-date guidelines should be followed for managing breast lumps,

## 1 Introduction

Breast cancer (BC) forms the highest proportion of cancer cases in both pre- and post-menopausal women worldwide [1] and is the commonest malignancy amongst Pakistani and Asian women [2]. However, it is extremely rare in the pediatric population; amounting up to 0.1 % of the total breast cancers in the US [3]. About 5–10 % of BC cases diagnosed worldwide are hereditary and confer a genetic mutation [4] and an early age of cancer diagnosis usually implies an ‘inherited cancer predisposition syndrome’ [5]. For breast cancer, the most important mutations include the BRCA1, BRCA 2, and TP53 genes which confer greater than a 10-fold increase in the relative risk of BC [6]. BRCA genes are important for cell cycle and transcriptional regulation, ubiquitylation, and DNA and damage implicates higher chances of cancer [7]. Lifetime risk of 60–70 % for the development of BC and 44 % risk for ovarian cancer is seen amongst BRCA mutation carriers. BRCA-positive Breast Cancer (BRBC) patients, additionally, have a >50 % risk of developing a second primary BC given that the first BRBC had developed at an early age [8]. As late detection in carriers causes worse patient outcomes [7], [9]. Thus, the National Comprehensive Cancer Network (NCCN) has come up with surveillance guidelines along with risk-reducing surgeries [8] for the patients and their family members.

As a staggering majority of pediatric breast masses are benign (mostly fibroadenoma) many a surgeon does not readily perform, on suspicion, a biopsy as it can potentially lead to a future breast disfigurement [3], [10]. However, this should not be standard practice [11]. In Pakistan, where there is a shortage of specialist doctors in rural areas misdiagnosis of rare diseases is a common occurrence. Surgeons not adequately trained in breast surgery tend to perform an initial excisional biopsy when the suspicion is intriguingly high while not performing adequate initial pre-operative workup. As James et al. further elaborate – such a practice can cause a delayed diagnosis, and hence, the treatment of pediatric BC [10]; this was also seen in our patient's scenario.

Here, we report the case of a teenage girl diagnosed with BC, having a pathogenic BRCA 1 mutation, and being an index case in her family. To our knowledge, this is the youngest patient reported in our country. It is of great emphasis that all patients diagnosed with BC aged under 50 years should be offered a cancer genetic risk assessment (including the consideration for testing and genetic counseling), however, only a few doctors do so. In Pakistan, there are only a handful of institutes that offer genetic counseling and testing facilities. This work has been reported in line with the SCARE criteria [12].

## 2 Case history

A 15-years old female, menarche at 11 years, presented to a clinic in her village in the Chagai district of Balochistan with the complaint of a right-sided breast lump for the last 6 months. Owing to the lack of diagnostic facilities in the village, a clinical diagnosis of fibroadenoma was made for which she electively underwent an upfront lumpectomy of the right upper-outer quadrant mass leading to a slight contour deformity. Post-operative histopathology showed a metaplastic carcinoma with heterogeneous differentiation. The size of the lesion was 6.5 × 6 × 3 cm and the tumor was reaching the outer painted margin. Cytokeratin AE1/AE3 were positive (EMA patchy positive, p63 patchy positive) while CK CAM 5.2 and 34-B-E12 were both negative. Thus, an incidental diagnosis of poorly differentiated, grade III, metaplastic breast carcinoma with mesenchymal differentiation was made and the patient was referred to our tertiary care hospital for further expert management.

**Fig. 1:**
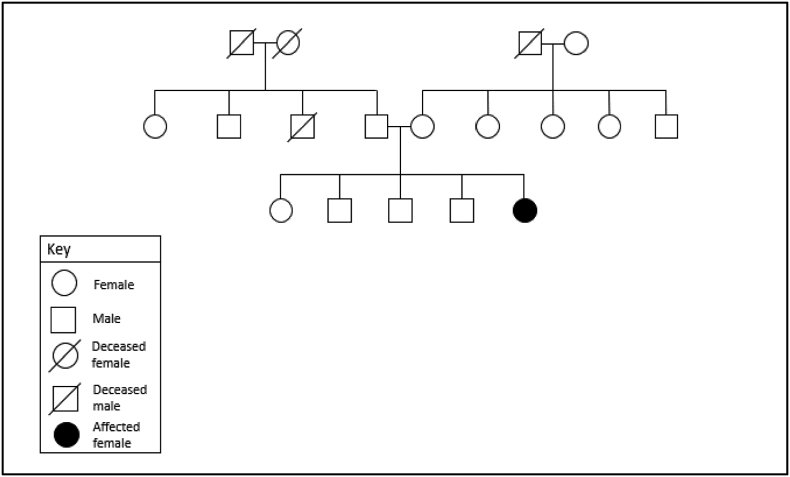
Patient's family tree.

At our hospital, further workup was carried out. A bilateral breast mammogram showed both the breasts as heterogeneously dense (BIRADS-0). In a subsequent ultrasound, the right breast was reported to be BIRADS-III with post-surgical changes of residual disease, and the left breast was identified as BIRADS-I. An MRI was also performed post-operatively which showed post-surgical changes. This was followed by a bone scan and a computed tomography (CT) of the chest, abdomen, and pelvis which were all unremarkable for any metastatic disease. A hormone receptor study performed showed a triple (ER/PR/Her2Neu) negative status. Family history revealed that the patient is the only child of her parents and is an index case of hereditary breast and ovarian cancer (HBOC) in her family as no first-degree relative has a history of breast or ovarian cancer (Fig. 1) and any other malignancy. Based on the history and test results, the utility of running a genetic test on the patient was considered for detecting a possible genetic mutation. The genetic test was performed using genomic DNA; sequence analysis and deletion/duplication testing were performed for 37 genes included in the: [1] ‘Invitae Breast and Gyn Panel’ and [2] ‘Add-on Preliminary-evidence Genes for Breast and Gyn Cancer’ [13]. Two heterozygous mutations were identified: [1] a loss-of-function pathogenic variant [c.1961dup (p.Tyr655Valfs*18)] of the BRCA1 gene linked with HBOC, also known as 2080insA in literature; and [2] a mutation of unknown significance in SMARCA4 gene [c.4845G > C (p.Glu1615Asp)]. The results confirmed a genetic cause of HBOC.

As the patient is a young unmarried girl, she and her family decided to conserve the breast with high-risk surveillance for breast and ovaries rather than embarking upon a bilateral risk-reducing mastectomy followed by breast reconstruction and risk-reducing salpingoopherectomy (RRSO). The family was counseled about the pros and cons of the available options and the risks of delaying a definitive surgery; hence, delaying the procedure was an informed decision.

Parenchymal distortion was seen at the surgical site leading to an obscured sentinel lymph node (SLN) on SLN mapping. Therefore, the patient underwent a re-excision and axillary clearance to get clear surgical margins. The surgical incision was the same as the earlier excisional biopsy and it did not cause any extra contour deformity of the 34C breast. The operating surgeon was an expert reconstructive breast surgeon. The obtained breast tissue sample exhibited foreign body giant cell reaction, fibroadenomatous changes, and adenosis, suggestive of a previous lumpectomy cavity site. No residual disease was identified in the submitted fibrotic area. All 11 lymph nodes showed reactive changes and no metastases were identified. This made the patient's status stage IIb, pT3N0M0.

Currently, she is on adjuvant chemotherapy, receiving Adriamycin + Cytoxan followed by weekly Carboplatin-Taxol, and planned for radiation therapy to the breast. The patient is also undergoing specialized surveillance with regular breast exams (more than once per year), an ultrasound pelvis, CA-125 levels, referral to a genetic counselor, yearly mammogram, and breast MRI in accordance with the recommendations set forth by NCCN [14], [15], [16]. The patient was also referred to a fertility counselor in case the patient and her family agreed upon RRSO later on in life; so that the patient and her family can take a timely and informed decision. Referral to a genetic counselor along with genetic testing has been advised to the first-degree relatives of the patient as advised by NCCN [16]. However, even with a confirmed genetic cause of the disease no individual from the patient's family has undertaken early screening for breast cancer. The presumed cause for this is a cultural taboo associated with cancers in the patient's vicinity.

## 3 Discussion

To the best of our knowledge, we herein report a case of the youngest known BRCA1 mutation BC in a patient aged 15 years from Pakistan. Studies have shown that younger patients with breast cancer exist in our population [17], [18]; however, no study looks at the specific characteristics of such patients. There is also a dearth of published data on HBOC amongst Pakistani pediatric BC patients.

Childhood cancer and, hence, Pediatric BC is termed a rare disease by the U.S. Rare Diseases Act of 2002 [19] as pediatric BC comprises <0.1 % of all BC and <1 % of all childhood cancers [20], [21]. Similar numbers have been seen in Pakistan, where a tertiary care hospital reported that 0.23 % of the cancer cases were BC in patients <20 years of age [18]. Another study from Lahore reported that childhood (0–14 years) and adolescent (15–19 years) BC amounted up to 0.03 % of total cancer incidences and 0.4 % of all childhood and adolescent cancer incidences in Lahore [17]. However, the mean age at onset for BC in the Asian population, when compared to the West, is much younger and there is a higher proportion of hormone receptor-negative patients [22], [23]. Hence, with almost a similar incidence the disease pathophysiology in the Asian population might differ slightly.

Germ-line mutations in the BRCA1/2 genes predispose women to HOBC. HBOC patients in the Pakistani population have a higher positivity rate of BRCA1/2 variants in their gene pool [24] with BRCA 1 mutations being a significant contributor to the high prevalence of BC in Pakistan (found in approximately 12 % of women with early-onset BC) [24], [25], [26], [27], [28], [29]. A BRCA 1 mutation was seen in our patient as well. BRCA1/2 genes produce growth suppressor proteins and mutation in any one of these two genes hinders DNA damage repair, leading to the accumulation of more mutations in cell DNA ultimately leading to cancer. Therefore, women with BRCA mutations have an average lifetime risk of BC of about 60 % [22]. Due to these effects, BRCA gene mutations are considered at high risk for the development of BC, as has also been observed in multiple case reports. However, when compared to the age of our patients, they were all much older [1], [3], [9], [22], [23].

BC in young women is of aggressive biological nature and has a propensity to be of already high grade at the time of diagnosis with a high proliferation index measured by Ki-67 nuclear expression and recurrence [10]. In contrast to late-onset BC features, early-onset BC exhibits the clinical-biological characteristics of aggressive disease because this type of BC has a greater frequency of being hormonal receptor/HER2-negative [10], [30]. The age at diagnosis is an imperative factor for prognosis and treatment decisions in patients with BC since diagnosis at a young age is typically linked with a less favorable prognosis [5]. The aggressiveness could partly be due to young women more often being diagnosed at advanced stages due to limited screening [5]. Furthermore, early-onset BC might respond less to therapy than the types of cancer seen in older women which might vary in their responsiveness to treatment, which is mostly hormonal manipulation. Thus early-onset BC shows a worse prognosis and outcome, despite aggressive treatment modalities [7], [9]. Therefore, early screening, detection, and subsequent treatments for patients with BC are vital to ensure improved patient outcomes. While our case report is the first of its kind to have detected an early onset BRCA positive BC; the prognosis is yet to be seen. The current success of the treatment modalities given to her offers an optimistic outlook for her future.

In line with the latest NCCN Guidelines for Breast Cancer Screening and Diagnosis, an ultrasound should have been performed for a clinically suspicious palpable breast lump in the initial evaluation of this patient (as the patient is aged <30 years). This should have been followed by an ultrasound-guided core biopsy if the ultrasound results resonate with the suspicion [11]. Diagnosis of metaplastic carcinoma was made based on histology; with benign-appearing axillary lymph nodes on US the patient was labeled as T3N0M0. Hence, she should have been followed with neoadjuvant chemotherapy succeeded by surgery and adjuvant radiation [31]. According to the guidelines, hormonal receptor status and Her2 status should also have been determined preoperatively [31]. Genetic testing should be performed in all patients diagnosed with breast cancer who are aged ≤45 years and all patients with triple-negative BC regardless of age [16]. The type of surgical procedure undertaken could have either been breast conservation surgery or mastectomy. However, the breast surgery would have probably remained the same given the family's wishes. In our case, the patient underwent an upfront excisional biopsy as a first diagnostic step rather than non-invasive imaging followed by a rather less invasive core biopsy. This does not follow the algorithm outlined by NCCN [16] and can lead to a disfigurement of the breast, additional surgical procedures, and increased morbidity. Hence, a lumpectomy should not be performed upfront without a staunch diagnosis.

Our team does not believe that the delay in presentation could have been prevented by surveillance as practiced in developed countries. This is because NCCN recommends screening in the general population from the age of 40 onwards or at age 5–10 years before the age of presentation of BC in the index case. This would mean that early diagnosis of this patient by surveillance could only have been possible if a first-degree relative aged 20–25 years had a BC. A delay in the presentation can be accredited to less awareness regarding breast cancer, delayed referral, and lack of available appropriate healthcare facilities in the region. This does not absolve developing an organized nationwide efficient screening and awareness campaign against BC. As Sidra et al. stated that the barriers to diagnosis and treatment of breast cancer can be eradicated by raising awareness for BC, educating the community on breast-self-examination, and community mobilization for breast cancer treatment [32].

Cancer care in Pakistan is expensive for the populace. The direct and indirect costs increase immensely if the patient has to get state-of-the-art care which is available in a rather distant private tertiary care hospital. Hence, the patient is being supported by Balochistan Awami Endowment Fund (BAEF). BAEF is an initiative of the Balochistan provincial government in which the government provides “impartial and need-based service delivery” in the form of “free of cost medical assistance to the needy patients of Balochistan” [33]. Hence, the patient's family has to bear the traveling cost only. The follow-up plan is managed such that it aligns with most other follow-ups at the hospital.

Cultural taboo against BC is an important socio-cultural phenomenon that can delay the initiation of diagnosis and treatment of BC in Pakistan [32]. Furthermore, due to the lack of established referral pathways and awareness of breast-self-examination in the country, the referral of BC patients can be delayed [34]. As highlighted by a Pakistani study, <70 % of patients presenting to a general practitioner with signs of BC were referred to a breast specialist, on average, after 30 days of initial presentation [34]. This delay in referral can be because general practitioners (GPs) are not sufficiently trained to diagnose complicated diseases like cancers [35]. This opens doors for discussion regarding the learning in LMICs for the optimum care of BC patients. At an individual level, the general population should be made aware of the disease: its presentation, its treatment options, and the disease progression. Targeted eradication of BC related stigma should take place via socio-cultural reforms; these reforms include increased research activity, policy changes, and education of the masses. There are only a handful of cancer support groups in Pakistan and none of them are dedicated to young patients with breast cancer such as the ‘The Young Survival Coalition (www.youngsurvival.org)’. Having such groups in Pakistan will help reduce the stigma and improve representation for patients who undergo misdiagnosis. The formation of local guidelines can help improve targeted BC care as it will take into consideration local culture and disease pathology. Furthermore, the establishment of clear and sturdy referral pathways should be targeted to prevent any delay in specialist referral. The GPs should be updated with the latest guidelines and have refresher courses for diseases that are seemingly less prevalent in their region so that overall care is improved. We also believe that to raise awareness for a rare disease and its appropriate management there should be more studies from our part of the world.

## 4 Conclusion

Although early-onset BC constitutes a small amount of the total BC incidence, it has a momentous burden on society. There is clear evidence that early-onset BC has a less favorable prognosis, and is more aggressive and intricate in its biological characteristics. Unquestionably, it is of prime importance to improve our understanding of the genetic predisposition and testing in lower-middle-income countries. Considering the changing global trends, as seen in our patient, we suggest that the utilization of genetic services is direly needed to improve the preventative care for at-risk individuals with breast and other cancers. LMICs need the delivery of BC awareness and self-examination campaigns amongst teenagers and adolescents in rural and urban areas to prevent a delay in diagnosis. The primary physicians in rural areas of Pakistan tend to deal with a very diverse range of diseases and so they should be taught about the initial management and timely referral of breast cancer to avoid a delayed presentation.

Learning points:

1. Every young patient diagnosed with breast cancer should have genetic testing.
2. Triple assessment is a mandatory tool to diagnose a breast lump in any age group.
3. Prophylactic procedures can be delayed with strict surveillance through counseling.
4. Genetic counseling should be readily offered in LMICs.
5. A lumpectomy should never be performed without a preoperative workup.

## Sources of funding

None.

## Ethical approval

This study was exempted from ethical approval at our institution.

## Consent

Written informed consent was obtained from the patient and her legal heir for publication of this case report. A copy of the written consent is available for review by the Editor-in-Chief of this journal on request.

## Research registration

This study is registered with Deutschen Register Klinischer Studien (ref). UIN: DRKS00029187.

(http://apps.who.int/trialsearch/<https://eur03.safelinks.protection.outlook.com/?url=http%3A%2F%2Fapps.who.int%2Ftrialsearch%2F&data=05%7C01%7Clubna.vohra%40aku.edu%7Cfd140406edd8420eb1b108da485b401c%7Ca5d4252a02f94e6096f09733baae4919%7C0%7C0%7C637901853999584585%7CUnknown%7CTWFpbGZsb3d8eyJWIjoiMC4wLjAwMDAiLCJQIjoiV2luMzIiLCJBTiI6Ik1haWwiLCJXVCI6Mn0%3D%7C3000%7C%7C%7C&sdata=LSOV1tHDwdFVZpxRWIYJA0I3CRgR%2BD3YfZIhEohqeDY%3D&reserved=0)

## Guarantor

Dr. Lubna Vohra.

## Provenance and peer review

Not commissioned, externally peer-reviewed.

## CRediT authorship contribution statement

Dr. Lubna Mushtaque Vohra – Conceptualization, investigation, resources, supervision

Dr. Danish Ali – Writing (original draft), writing (review and editing), corresponding author

Dr. Syeda Amrah Hashmi – Writing (original draft), data collection

Ms. Meher Angez – Writing (original draft)

## Declaration of competing interest

None.
